# Challenges in Therapeutically Targeting the RNA‐Recognition Motif

**DOI:** 10.1002/wrna.1877

**Published:** 2024-12-12

**Authors:** Stefan Schmeing, Peter 't Hart

**Affiliations:** ^1^ Chemical Genomics Centre of the Max Planck Society Max Planck Institute of Molecular Physiology Dortmund Germany

**Keywords:** inhibitors, mRNA processing, RNA‐binding proteins, RNA‐recognition motif

## Abstract

The RNA recognition motif (RRM) is the most common RNA binding domain found in the human proteome. RRM domains provide RNA‐binding proteins with sequence specific RNA recognition allowing them to participate in RNA‐centric processes such as mRNA maturation, translation initiation, splicing, and RNA degradation. They are drivers of various diseases through overexpression or mutation, making them attractive therapeutic targets and addressing these proteins through their RRM domains with chemical compounds is gaining ever more attention. However, it is still very challenging to find selective and potent RNA‐competitors due to the small size of the domain and high structural conservation of its RNA binding interface. Despite these challenges, a selection of compounds has been reported for several RRM containing proteins, but often with limited biophysical evidence and low selectivity. A solution to selectively targeting RRM domains might be through avoiding the RNA‐binding surface altogether, but rather look for composite pockets formed with other proteins or for protein–protein interaction sites that regulate the target's activity but are less conserved. Alternative modalities, such as oligonucleotides, peptides, and molecular glues, are exciting new approaches to address these challenging targets and achieve the goal of therapeutic intervention at the RNA regulatory level.

## Graphical abstract

Targeting of RNA recognition motifs can modulate mRNA processing and provide new therapeutic strategies. Challenges in selectivity and potency suggest that alternative targeting strategies are required for efficient inhibition.

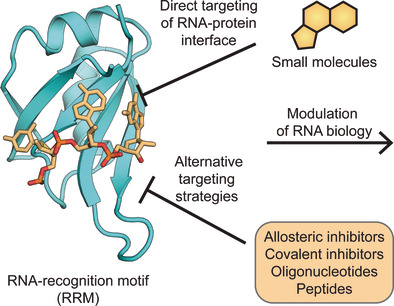

## 1 Introduction

**FIGURE 1:**
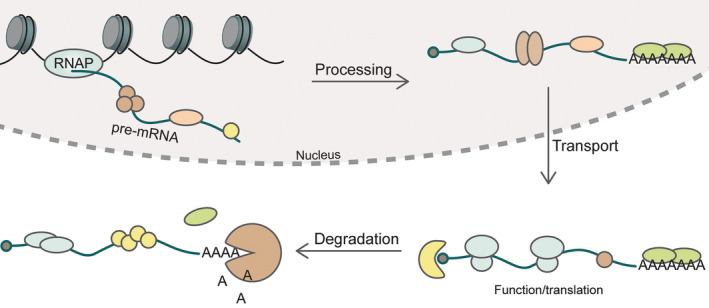
RNA is bound by a variety of RNA‐binding proteins during its lifetime. The proteins regulate the processing, transport, and degradation of their target mRNAs.

Although only a small part of the human proteome encodes for protein, a much larger part is actively transcribed (International Human Genome Sequencing Consortium 2001) The resulting coding and noncoding RNA does not act on its own, but is bound by RNA‐binding proteins (RBPs) (Birney et al. 2007; J. Cheng et al. 2005). Any RNA is bound by a range of RBPs that vary significantly during its lifetime and control its biogenesis, transport, function, and degradation (Figure 1) as highlighted in a recent study by Choi et al. (2024). These RBPs use various conserved domains to interact with RNA including the RNA recognition motif (RRM), K‐homology (KH), DEAD/DEAH helicase, and zinc‐finger domains (Gerstberger, Hafner, and Tuschl 2014), but RNA‐binding can also occur through domains that were not originally annotated for this purpose or via intrinsically disordered domains (Balcerak et al. 2019; Baltz et al. 2012; Castello et al. 2012). From all these domains, the most common RNA‐binding domain is the RRM, which is among the most abundant protein domains in the human proteome (Choi et al. 2024; Venter et al. 2001). In fact, it is present in 0.5%–1% of human protein coding genes and plays a crucial role in many biological pathways, but can also be found in the genomes of plants, bacteria, and viruses (Albà and Pagès 1998; Maris, Dominguez, and Allain 2005; Venter et al. 2001). Many roles for RRM containing proteins have been identified in eukaryotes including: splicing, alternative splicing, localization of proteins and RNAs, stability of RNAs, and regulation of translation, while the roles of such proteins in other organisms are less well studied (Maris, Dominguez, and Allain 2005).

Proteins with RRM domains are commonly involved in RNA regulation and disease making them possible therapeutic targets. Especially their role in alternative splicing is of therapeutic interest since aberrant splicing regulation can lead to a variety of diseases including cancer, neurodegenerative, metabolic, cardiovascular, and immune diseases (Y. Zhang et al. 2021). Comparison of cancerous tissue with healthy tissue has shown there are thousands of cancer‐specific splicing patterns caused by aberrant splicing factor proteins (Brooks et al. 2014; Ferreira et al. 2014; Furney et al. 2013; Graubert et al. 2012; Jung et al. 2015; PCAWG Transcriptome Core Group et al. 2020). Often, the affected transcripts play a role in the hallmarks of cancer and thus it can be assumed that the deregulation of splicing induces disease phenotypes (Bechara et al. 2013; Ben‐Hur et al. 2013; Dominguez et al. 2016; Pradella et al. 2021; L. Wang et al. 2016). The defects causing such splicing changes are commonly caused by changes in the levels or mutation of splicing factors and especially proteins of the SR and heterogeneous nuclear ribonucleoprotein (hnRNP) families are often found to be oncoproteins or tumor suppressors (Frampton et al. 2015; Golan‐Gerstl et al. 2011; Jbara, Siegfried, and Karni 2021; Karni et al. 2007; S. C. W. Lee and Abdel‐Wahab 2016). For example, mutations in the RRM domain of the splicing factor SRSF2 affect RNA binding and are observed in myelodysplasia and various types of leukemia (Lindsley et al. 2015; Patnaik et al. 2013; Yoshida et al. 2011). Furthermore, increased levels of RRM containing proteins in cancer is illustrated by the overexpression of the splicing factors Polypyrimidine tract‐binding protein 1 (PTBP1) in glioma and hnRNP A2/B1 in breast cancer (Klinge et al. 2019; Zhu et al. 2019). Both proteins can influence the alternative splicing of pyruvate kinase which regulates cancer cell metabolism (Clower et al. 2010). Besides cancer, RRM containing proteins are involved in neurodegenerative diseases where the single RRM domain containing FUS and the dual RRM containing Tar DNA‐binding protein 43 (TDP‐43) are both implicated in amyotrophic lateral sclerosis (ALS), while the latter protein is also involved in frontotemporal lobe degeneration (Arai et al. 2006; Kwiatkowski et al. 2009; Neumann et al. 2006; Suk and Rousseaux 2020). Based on all these pathological roles of RRM containing proteins, the interest in targeting RRMs for therapeutic treatment has been increasing in the recent years, and the main goal of most inhibitors is to inhibit RNA binding. In the following section, we will first introduce the RRM domain from a structural perspective followed by a discussion of examples of small molecules and other modalities designed to inhibit RRM–RNA interactions.

### 1.1 Structure of the RRM

**FIGURE 2:**
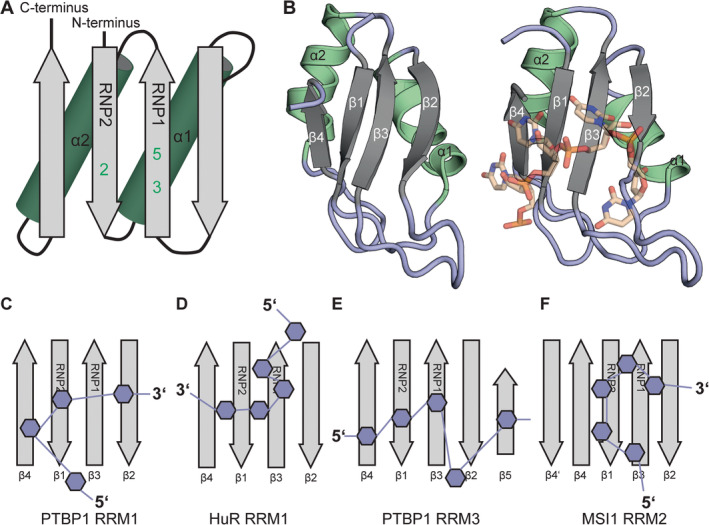
(A) Schematic representation of the RRM domain. The numbers indicate the relative positions of the aromatic residues that drive RNA‐binding. (B) Left: RRM1 of *Drosophila melanogaster* sex‐lethal in apo form (PDB ID: 2sxl). Right: RRM1 of sex‐lethal in RNA‐bound form (PDB ID: 1b7f). (C–F) Schematic representation of the variation in RNA‐binding poses on the β‐sheet surfaces of several RRMs.

The RRM is a small domain consisting of approximately 90 amino acids which binds single stranded nucleic acid (RNA and DNA) and can facilitate protein‐protein interactions (PPIs) in bigger protein‐RNA complexes (Cléry, Blatter, and Allain 2008). The RRM folds into a conserved β_1_α_1_β_2_β_3_α_2_β_4_ topology (Figure 2A) consisting of a beta‐sheet of four beta strands stacked by two helices (Nagai et al. 1990). Both helices are located on the “back” of the sheet, while the “front” acts as the RNA binding site. In the center of the flat RNA binding site are three aromatic residues that are the main drivers for nucleic acid binding referred to as the RNP residues (Cléry, Blatter, and Allain 2008). The first is the second residue of β_1_ (also known as RNP2), while the other two are the third and fifth residue of β_3_ (also known as RNP1) (Figure 2A,B). Due to the high conservation of the RNP motifs, the consensus sequences of the β_1_ and β_3_ strands were identified as I/L/V‐**F/Y**‐I/L/V‐X‐N‐L for RNP2 and R/K‐G‐**F/Y**‐G/A‐**F/Y**‐I/L/V‐X‐F/Y for RNP1 (X: any amino acid; bold amino acids are key residues) (Afroz et al. 2015). The RNA typically lies over the β‐sheet as exemplified by the first RRM of the *Drosophila melanogaster* sex‐lethal protein in Figure 2B. The lower conservation of the rest of the domain allows for the RNA to be positioned in various orientations on the sheet with varying positions of the individual nucleotides (Figure 2C–F) (Daubner, Cléry, and Allain 2013). The variety of binding modes is not only caused by the less conserved residues in the sheet, but also by additional secondary structure elements that are commonly observed for RRMs (Afroz et al. 2015). Frequently, these come in the form of extensions of the sheet as additional strands or as flanking helices (Figure 2E,F). In many cases, additional strands allow for an additional nucleotide to be bound, but in other cases, the extensions can promote PPIs or intramolecular interactions stabilizing specific elements of the protein fold. An example of such an extended RRM is RRM3 of PTBP1, which is extended by a fifth β‐strand and thus can bind five nucleotides, each on one strand (Figure 2E) (Conte 2000). While MSI1 RRM2 is extended through a β4′ strand, this RRM does not utilize the extra space for RNA binding with five nucleotides binding on β3, β1 and β2 (Figure 2F) (Iwaoka et al. 2017). More complicated are the roles of additional alpha‐helices on the termini the RRM domain. Similar to the β‐strand extensions, helices can enlarge the binding site and facilitate binding to RNA directly. Alternatively, N‐ or C‐terminal helices flanking the RRM can influence RNA binding indirectly through allosteric effects or through the recruitment of effector proteins (Afroz et al. 2015). RRM1 of PTBP1 is extended through a transient α3 helix, which folds upon the RRM surface upon RNA binding. By this, the affinity for the RNA is improved although the helix does not make direct RNA contacts (Maris et al. 2020). An extensive integrative structural biology approach demonstrated that this helix plays an important role in intramolecular interactions organizing the tertiary structure of PTBP1 bound to an RNA (Damberger et al. 2024; Dorn et al. 2023). Further examples are RBM39 RRM1 and RRM2, which both have a C‐terminal helical extension and even though the helix itself does not contact the RNA, the loops that connect the helices to the RRMs do (Campagne et al. 2023).

Another level of complexity in the interaction between RNA and RRM containing proteins is added through the presence of multiple RRM domains. Although there are several proteins with a single RRM, many others have more, allowing them to bind longer RNA sequences and thus achieve higher binding affinity and selectivity (Afroz et al. 2015). The human dead end 1 protein contains two RRM domains and while RRM2 can't bind nucleic acids alone, it increases RRM1s affinity up to 80‐fold from 54 to 0.86 μM for 7–8 nt long sequences (Duszczyk et al. 2022). RNA binding is driven by RRM1, but is sandwiched by both domains in a positively charged channel stabilized by RRM2. A similar tandem mode of action by sandwiching RNA was observed for the protein HuR where the RNA binds in a positively charged cleft between RRM1 and RRM2 (H. Wang et al. 2013). Other multi‐RRM proteins orchestrate RNA binding with two domains differently. RRMs 3 and 4 of PTBP1 as well as RRMs 1 and 2 of hnRNP A2/B1 bind RNA in a more linear fashion via the β‐sheet, with an individual consensus sequence per RRM unit. In both cases, a stiff conformation of the RRMs toward each other organizes the RNA fold, which is necessary for the biological function in splicing regulation (Oberstrass et al. 2005; B. Wu et al. 2018). Not all RRMs of multi‐RRM proteins work together, but can also act independently of each other or in combination with non‐RRM RNA‐binding domains (Y. Chen et al. 2020; Crowder et al. 1999). The existence of multiple domains in a single RBP complicates the search for potent inhibitors, as inhibition of a single domain might not be sufficient for a global blockade of its RNA‐binding capacity.

**Sidebar Title: Other RNA‐Binding Domains**

Besides the RRM domain, there are various other canonical RNA‐binding domains found in the human proteome. Those with folded secondary structures include the KH domain (Valverde, Edwards, and Regan 2008), the DEAD box helicase (Linder and Jankowsky 2011), zinc‐finger (Cassandri et al. 2017), and the Pumilio domains (Nishanth and Simon 2020) among various others that occur less frequently (Gerstberger, Hafner, and Tuschl 2014). Besides structured domains, there are also various intrinsically disordered domains that do not have a stable fold in the unbound state, but might form specific secondary structures when bound to RNA (Zeke et al. 2022). The domains that form helices when bound to RNA are typically rich in arginines, while those that form loops are rich in glycine and proline. Most RBPs use a combination of such domains to selectively interact with their targets and achieve high affinity (S. Liu et al. 2020).

### 1.2 How to Target an RRM?

Targeting the RRM domain can be done via two strategies. The RNA‐binding interface can be targeted directly, but due to the high conservation of this interface, achieving selectivity is challenging. The globular fold of the domain lacks well defined and hydrophobic pockets making potency hard to achieve as well (Walters et al. 2023). Alternatively, binding sites away from the RNA‐binding interface could be targeted, which typically are less conserved and might therefore lead to more selective inhibition. Nonetheless, these interfaces might not always be relevant for the desired therapeutic approach and only inhibit part of the function of the target protein.

Besides the mode of binding, the selection of the used chemical matter is also of importance. Due to the limited availability of protein‐RNA interaction inhibitors, it is hard to judge what chemical space should be explored. The lack of defined pockets, as well as the large size of the interface to be inhibited, make it likely that the rules for potent inhibition follow those of protein–protein interaction inhibitors that address similar situations (Arkin, Tang, and Wells 2014). The distribution of hot‐spot residues (those that contribute most to RNA‐binding) on the RNA‐binding interface is another factor to take into account and based on the diverse RNA‐binding modes described above it is clear that these can be far apart. To address such a wider distribution, it is conceivable that larger molecules are required (Egbert et al. 2019).

Although most described approaches have used small molecules, alternative modalities such as oligonucleotides and peptides have been explored as well. Due to their larger size, they can interact with larger parts of the RRM surface by making more specific contacts and potentially providing them with higher potency and selectivity for their target. However, these alternative modalities do typically suffer from limited membrane permeability, which is essential for reaching intracellular targets such as RRM containing proteins. Their stability in biological systems might also pose a challenge, but for both problems modern chemical solutions are available.

### 1.3 Direct Targeting of the RNA‐Binding Interface of RRMs With Small Molecules

Although the RRM domain could be considered “undruggable” from a traditional point of view, a variety of small molecule ligands have been described originating from synthetic libraries, natural product libraries and virtual libraries. Here we will describe such inhibitors categorized by the targets they were identified for. The overview is limited to those molecules with biophysical evidence for interacting with an RRM domain.

### 1.4 HuR

**FIGURE 3:**
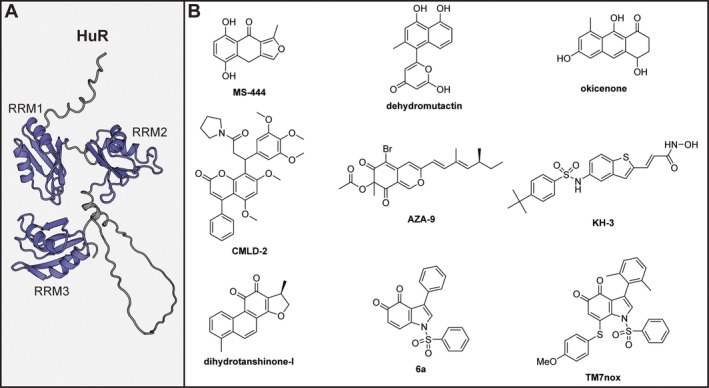
(A) AlphaFold predicted structure of full length HuR. RRM domains are shown in blue, unstructured domains in gray. (B) Chemical structures of HuR inhibitors.

The RBP HuR was one of the first to be targeted with small molecules in the early 2000s (Meisner et al. 2007). HuR is an essential factor for stabilization of AU‐rich elements (AREs) in 5′‐and 3′‐UTRs and many of the mRNAs regulated by HuR encode for genes involved in inflammatory processes, cell division, migration, and metabolism (Abdelmohsen et al. 2007; X. Guo and Hartley 2006; H. H. Kim et al. 2009; A. Lal et al. 2004; Saunus et al. 2008; Sheflin, Zou, and Spaulding 2004; Topisirovic, Siddiqui, and Borden 2009). HuR has three RRM domains and it is the first two that drive binding with AREs while the third regulates binding to poly‐A tails (Figure 3A) (H. Wang et al. 2013). HuR is a key regulator in cancer where it is often found to be overexpressed, but also thought to be overactive by its increased cytoplasmic localization (Schultz et al. 2020). It received a lot of attention in cancer biology as it mediates resistance against radiation, chemotherapeutics and other anticancer agents (Blanco, Jimbo, et al. 2016; Chand et al. 2017; Romeo et al. 2016). Therefore, its inhibition provides the opportunity for sensitization to these therapies.

The first small molecule inhibitors of HuR, **MS‐444**, **dehydromutactin**, and **okicenone**, (Figure 3B) were identified using an anisotropy based high‐throughput screening (HTS) assay using HuR and an ARE‐RNA (Meisner et al. 2007). The hits were obtained from a library of extracts of various organisms and active extracts were fractionated followed by identification of the compounds. The report mathematically defines a binding model where HuR interacts with RNA in a 2:2 stoichiometry based on anisotropy measurements. Using competition experiments and the defined model, the authors calculated that the compounds inhibit dimerization of HuR and RNA binding with nanomolar affinities. However, a large discrepancy is observed when the inhibitory potency against HuR dimerization in a gel‐based assay was measured where mid to high micromolar concentrations were required. Similarly, in cellular context, micromolar concentrations are required to observe changes in HuR localization and cytokine expression in primary human monocytes (Meisner et al. 2007). In follow‐up studies, the cellular effects of **MS‐444** were quantified in colorectal cancer cell lines (EC_50_ = 5–40 μM) and the mode of action was shown to be ARE and HuR dependent (Blanco, Preet, et al. 2016). Consistent with the cellular role of HuR, its inhibition leads to apoptosis in colorectal cancer cells. Furthermore, a shift in subcellular localization of HuR from the cytoplasm into the nucleus was observed upon **MS‐444** treatment and it increased sensitivity to the TNF‐related apoptosis inducing ligand (TRAIL) in pancreatic ductal adenocarcinoma (Romeo et al. 2016). It has to be noted that **MS‐444** was previously identified as an inhibitor of myosin light chain kinase, indicating that its cellular activity might be of a polypharmacological nature (Nakanishi et al. 1995).

**CMLD‐2** (Figure 3B) was the next HuR inhibitor identified by a fluorescence polarization (FP)‐based HTS campaign of approximately 6000 compounds (*K*_i_ of 0.35 μM) (X. Wu et al. 2015). It was further validated in an AlphaLISA assay, using HuR RRM1 and 2 where a *K*_i_ of 3.4 μM was measured and the hit showed higher anti‐proliferative effects in cell viability experiments with cancer cell lines in comparison to non‐cancerous cell lines. Furthermore, HuR bound mRNA levels were reduced in an RNA‐immunoprecipitation (RIP) assay and Wnt signaling was significantly affected upon **CMLD‐2** treatment. The same group later used the same FP assay to screen a further 2000 compounds and reported the identification of **AZA‐9** and **KH‐3** as novel HuR inhibitors (Figure 3B) (Kaur et al. 2017; X. Wu et al. 2020). Although **AZA‐9** was able to inhibit HuR in an FP assay with an IC_50_ of 1.2 μM, it was only further studied using NMR and docking studies which suggested it might bind to the RNA binding cleft. **KH‐3** was studied more in depth and found to have a *K*_i_ of 830 nM for the interaction between full length HuR and an Msi1 derived RNA sequence. Both FP and SPR were used to determine that the molecule targets both RRM1 and 2 and docking studies indicated it also bound the RNA‐binding groove. A cellular thermal shift assay (CETSA) showed that **KH‐3** could stabilize HuR in cells indicating it indeed engaged the target. By using RIP experiments, the authors showed that **KH‐3** was able to inhibit binding of HuR to various ARE containing mRNAs, while the negative control compound KH‐3B could not. **KH‐3** decreased the half‐life of these mRNAs while the control compound had no effect indicating that HuR regulated mRNA stability was inhibited. The compound had cytotoxic activity against a variety of triple‐negative breast cancer cell lines, although knock out of HuR did not affect the IC_50_ very strongly (2.6‐fold reduction). RNA‐sequencing (RNA‐seq) was then used to detect changes in gene expression upon **KH‐3** treatment, while RIP sequencing was used to explore which RNAs were bound by HuR. In the overlap between these two data sets, the authors identified FOXQ1 as a relevant target gene contributing to HuR driven cell invasion, which could be reduced by **KH‐3** treatment. The compound was also tested in a mouse MDA‐MB‐231 xenograft model, where it significantly reduced tumor size as well as lung metastasis and survival.

The first HuR inhibitor described that has low nanomolar activity is **dihydrotanshinone‐I** (**DHTS**, Figure 3B), which was identified in an HTS campaign using a library of 107 known anti‐inflammatory agents (D'Agostino et al. 2015). The molecule was found to have a *K*_I_ of 3.74 nM in an AlphaScreen assay for the association of HuR to an ARE‐RNA and was further validated using an electrophoretic mobility shift assay (EMSA). Truncation studies showed that the molecule inhibited RNA‐binding of a HuR variant containing both RRM1 and 2, but not of truncations that contained 2 and 3 or the individual domains. It must be noted that the concentrations required in the EMSA assays to achieve 50% inhibition are in the low micromolar range, rather than the nanomolar range. *In cellulo* experiments showed that **DHTS** reduced TNF mRNA levels (which has an ARE sequence) and was able to inhibit MCF‐7 cell growth with an IC_50_ of 0.84 μM by inducing apoptosis, as measured by caspase activity. However, knockdown or overexpression of HuR had only a moderate effect on the IC_50_ (0.45 and 1.30 μM, respectively), potentially indicating that the cellular effects of the compound are not caused by HuR inhibition alone. Indeed, when tested with another ARE‐binding protein (hnRNP D) inhibitory effects were observed as well. In a follow up report, **DHTS** was studied by NMR and MD simulations, which confirmed the interaction with RRM1 and 2 and binding of the RNA binding groove (P. Lal et al. 2017). Point mutations of the targeted residues led to a loss of inhibition, further confirming the binding region. However, RIP experiments analyzed by microarrays led to the contradictory observation that **DHTS** inhibited only 79 transcripts from binding by HuR but increased the interaction with 558 transcripts. Although **DHTS** did seem to have an effect on tumor growth in mice, it is still an open question how it elicits this effect since various other targets have been described for **DHTS** such as topoisomerase I and HIF‐1α (X. Chen et al. 2019).

The inhibitory activity of **DHTS** was increased fivefold in the AlphaScreen assay, through a SAR study around the *o*‐benzoquinone core, yielding compound **6a**. A combination of NMR experiments and MD‐simulations lead to the hypothesis that **6a** stabilizes HuR RRM1 and 2 in a closed conformation, which is incompatible with RNA binding, in line with the truncation studies on **DHTS** (Manzoni et al. 2018). In cellular assays, the new molecule was found to be less active than **DHTS** itself, further highlighting that this parent molecule likely has multiple targets. The same scaffold was further optimized to yield **TM7nox** (Figure 3B), which has an IC_50_ of 800 nM in an homogeneous time resolved fluorescence assay. **TM7nox** was shown to have a similar mode of action, binding to the same pocket as **6a** and **DHTS** (Bonomo et al. 2023). RNA‐seq analysis showed that **TM7nox** was able to reduce the interaction between HuR and several LPS induced genes.

Although other molecules were reported to modulate HuR in various ways, their exact binding modes and therefore the possible interaction with its RRM domains were not investigated (Y.‐C. Cheng et al. 2013).

### 1.5 Musashi 1 and 2

**FIGURE 4:**
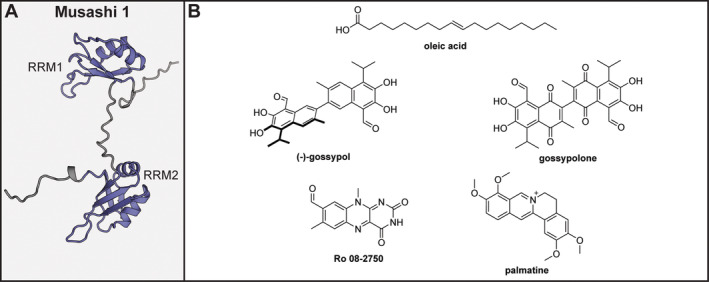
(A) AlphaFold model of Musashi 1 (residues 1–200). RRM domains are shown in blue, unstructured domains in gray. (B) Chemical structures of Musashi 1 and 2 inhibitors.

The family of Musashi proteins was first described in 1994, where they were found to play a role in the regulation of division of *Drosophila melanogaster* sensory organ precursor cells (Nakamura et al. 1994). In humans, there are two members to this family, MSI1 and MSI2 which share a 75% sequence homology, that are thought to guide the differentiation of normal cells and neuronal progenitors as well as play a role in organ development (Ito et al. 2010; Kharas et al. 2010; Sakakibara et al. 2001). In 2001, they were first linked to cancers, and by now their upregulation has been discovered in gliomas, medulloblastomas, hepatomas, and various forms of leukemia (Barbouti et al. 2003; Fox et al. 2016; Gao et al. 2015; K. Guo et al. 2017; Hemmati et al. 2003; Kagara et al. 2012; Kanemura et al. 2001; Kang et al. 2017; Kudinov et al. 2016; J. Lee et al. 2016; Oskarsson et al. 2011; Shu et al. 2002; X.‐Y. Wang et al. 2010; C. Yang et al. 2016). Both human Musashi proteins have two RRMs, and for MSI1 a PPI domain (LD) and a poly‐A‐binding domain were identified (Figure 4A).

The first molecule inhibiting MSI1 (**oleic acid**) was identified in a classical high throughput screen of 30,000 compounds by Clingman et al. using a FP‐based assay (Clingman et al. 2014). Although a few more traditional small molecule hits were identified, the authors chose the hit **oleic acid** (Figure 4B) for further studies which inhibited MSI1 binding to RNA with a *K*_i_ of 1.2 μM. Other unsaturated fatty acids were able to inhibit MSI1 in the same affinity range, and a conformational change in RRM1 causing a loss of RNA binding capacities was proposed. An *in silico* model predicted an allosteric hydrophobic pocket on the α‐helical side of RRM 1, opposite to its RNA‐binding residues, which could be confirmed by mutagenesis. Treatment of rat oligodendrocyte progenitor cells with **oleic acid** led to reduced proliferation and the authors identified a feedback loop of ω‐9‐fatty acid biosynthesis and MSI activity. The identified fatty acids also bound to MSI2, but were not tested against other proteins of the RRM family.

One year later, a natural product, **(−)‐gossypol** (Figure 4B), was identified as a MSI1 inhibitor again via an FP‐based HTS campaign, which inhibited MSI1 RNA binding with a *K*_i_ of 476 nM. Via truncation of MSI1, the authors identified the first RRM domain as the site of interaction, although the second RRM was not tested (Lan et al. 2015). SPR analysis further indicated interaction with RRM1, albeit with weaker affinity than measured by FP, and peak shifts observed during NMR analysis suggest the molecule directly binds the RNA binding site. The compound was tested for its ability to inhibit viability of various cancer cell lines, but also those with low MSI1 expression were affected, possibly indicating that the mode of action is not selective for MSI1. In a follow‐up study, the authors investigated the analogue **gossypolone** (Figure 4B) (Lan et al. 2018). The molecule was determined to have a *K*_i_ of 12 and 7 nM in a competitive FP screen between a *numb* RNA probe and full length MSI1 and MSI2, respectively. However, SPR analysis demonstrated no response at a 2.5 μM concentration.

In two more recent reports by Minuesa et al., the MSI2 inhibitor **Ro 08‐2750** (Figure 4B) was described, after identification using an FP‐based HTS campaign of a library of 6208 compounds (Minuesa et al. 2014, 2019). The molecule was found to have an IC_50_ of 2.7 μM in an FP assay for MSI2 RNA‐binding, which could be confirmed using EMSA. The *K*_D_ for the target protein was determined using microscale thermophoresis (MST) and found to be 12.3 μM. Interestingly, MST was used to measure the affinity of the molecule for several other RRM containing proteins including SYNCRIP, SRSF2, HuR, RBMX, and TIA‐1. Although **Ro 08‐2750** bound to all other proteins, except for HuR, the affinities were relatively weak with the highest affinity observed for SRSF2 (190 μM). A docking model indicated that the inhibitor directly interacts with the RNA‐binding residues of RRM1 and mutation of any of these residues led to reduced affinity confirming the binding site hypothesis. **Ro 08‐2750** was able to reduce viability of various leukemia cell lines, although the EC_50_ for some cell types were lower than the affinity for MSI2. The discrepancy indicates that the effect might be caused by interaction with other targets, such as the NGF receptor for which it was originally described (Niederhauser et al. 2000). Leukemia models in mice did not show a change in disease progression, but did show some improvements in certain indicators. A more recent study investigated **Ro 08‐2750** in a proteome wide manner using a thermal proteome profiling experiment (Walters et al. 2023). Of the 768 proteins that the molecule interacted with, 145 were known RBPs with RRM domains. Relative to these other RRM containing proteins, the interaction between **Ro 08‐2750** and MSI2 was weak. The authors further demonstrated that various phenotypes induced by the **Ro 08‐2750** were not related to MSI2, and that the molecule causes cellular stress at higher concentrations. This study is an essential indicator of the challenges of selective targeting of the RRM domain and highlights that proteome wide selectivity evaluation is key to understanding the mode of action of a given inhibitor.

Zhang et al. investigated the role of MSI2 in colon cancer via proteomics and transcriptomics approaches, comparing both wild‐type cells and MSI2 knockout cells (X. Zhang, Su, et al. 2022). After confirming MSI2 as a potential therapeutic target, both the previously described **Ro 08‐2750** and **(−)‐gossypol** were used to investigate whether inhibition led to the desired effect. However, both compounds were found to be equally effective in both wild‐type and MSI2 knockout HCT116 cells, again indicating that their activity is not related to MSI2 inhibition. Therefore, a new cell‐based high‐throughput screen was developed, based on cell growth inhibition of both HCT116 variants, so that the activity of a given compound could be directly compared. As a secondary screen, cell growth inhibition against another cell line (A549) was tested and again compared between both MSI2 wild‐type and knockout variants. Since the growth of wild‐type A549 cells does not depend on MSI2 no difference would be expected for an MSI2 inhibiting compound. A library of 1109 natural compounds was tested in this set‐up and four compounds were found to have the expected effect. The compound **palmatine** (Figure 4B) was further investigated using MST for binding to GFP‐MSI2 in cell lysate and found to have an affinity of 26.4 μM. The binding affinity of the compound for each RRM individually was then tested and binding was only observed for RRM2. Using the same assay set‐up, the compound was tested for binding to MSI1, HuR, and LIN28A and found to have similar affinities of 67.5, 33.5, and 49.6 μM, respectively. These findings indicate that the compound is not specific, especially since LIN28A does not have any RRM domains. Cellular assays did show the compound to be more effective in MSI2 wild‐type HCT116 cells in comparison to knockout cells but the effects might be indirectly related to MSI2 as it is a well‐known DNA damaging agent and has a wide variety of other targets (Long et al. 2019).

### 1.6 hnRNP A2/B1

**FIGURE 5:**
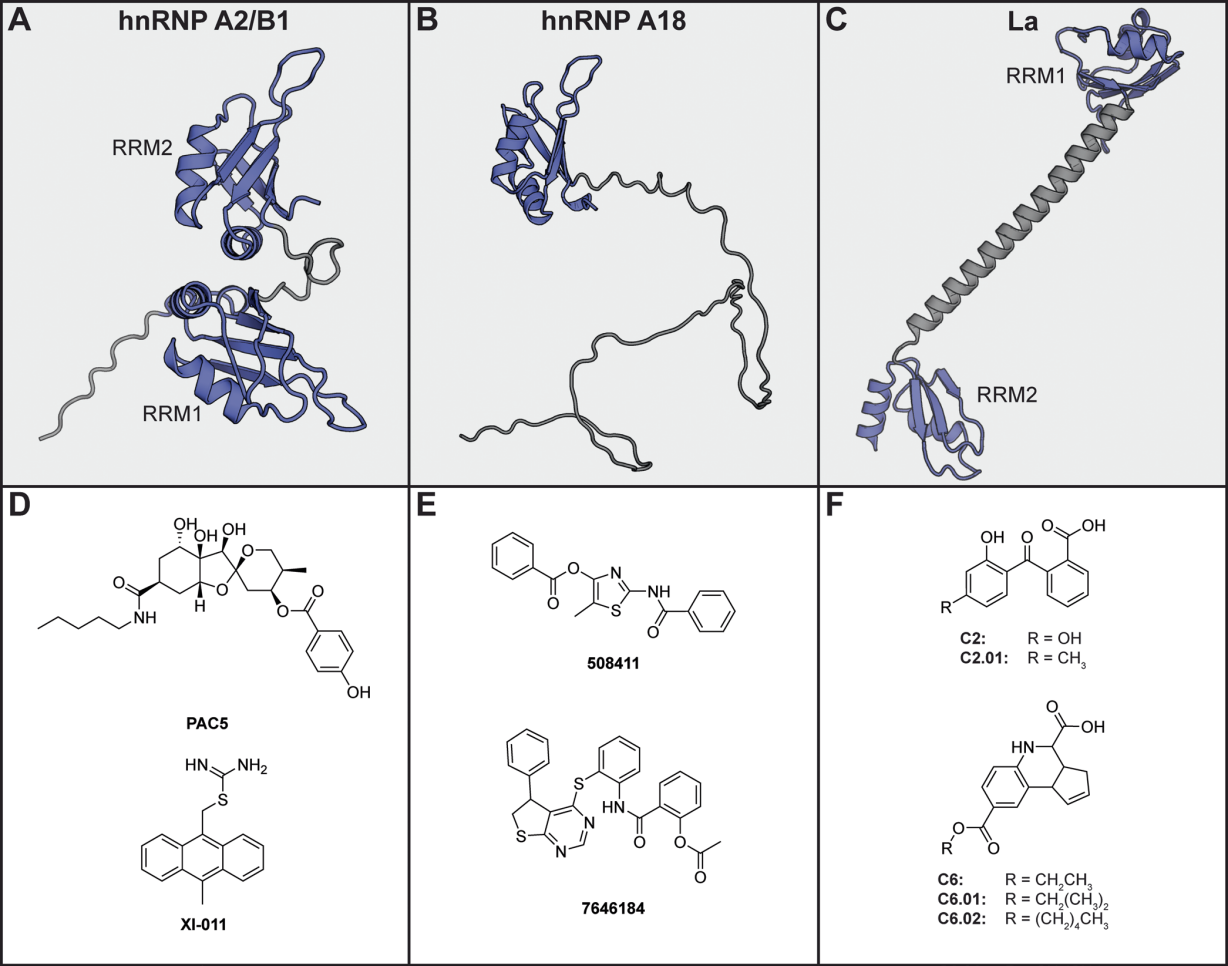
(A) AlphaFold model hnRNP A2/B1 (residues 1–220). (B) AlphaFold model of full length hnRNP A18. (C) AlphaFold model of La (residues 111–290). For (A–C), the RRM domains are shown in blue, unstructured domains in gray. (D) Chemical structures of hnRNP A2/B1 inhibitors. (E) Chemical structures of hnRNP A18 inhibitors. (F) Chemical structures of hnRNP La inhibitors.

The splicing factor hnRNP A2/B1 is part of the family of hnRNPs, which has several members with RRM domains. They tend to negatively influence splicing events near their binding sites on mRNA and by doing so lead to exclusion of nearby exons (Martinez‐Contreras et al. 2007). The structure of hnRNP A2/B1 contains two RRM domains followed by an unstructured prion‐like domain (Figure 5A) (B. Wu et al. 2018). Since it regulates the processing of a large number of mRNAs, it plays a role in a variety of diseases (Y. Liu and Shi 2020). It is often found to be upregulated in cancers, thereby leading to transcriptome wide splicing changes promoting proliferation, metastasis, and angiogenesis (Dai et al. 2017; Eswarappa et al. 2014; Y. Hu et al. 2017). Mutation or downregulation of hnRNP A2/B1 has been observed in neurodegenerative disorders such as Alzheimer's disease and ALS (Berson et al. 2012; H. J. Kim et al. 2013). Since downregulation seems to elicit negative physiological effects, it would be important to investigate unwanted side effects when applying therapeutic inhibitors for this protein.

The first inhibitor described for hnRNP A2/B1 was **PAC5** (Figure 5D), which is an analogue of the sesquiterpenoid phyllanthacidoid A (Zuo et al. 2023). It was identified from a library of natural products in a screen against hepatitis B virus followed by structural optimization of the alkyl chain (pentyl amide in **PAC5**). The compound was converted into a pull‐down probe by attachment of biotin and an analogue with a shortened propyl alkyl chain was used as a negative probe. The probes were used for a proteomics experiment leading to the identification of hnRNP A2/B1 as a possible target of **PAC5**. Docking analysis identified two pockets with good scores and based on these binding poses several mutations were made. The pull‐down was then repeated using the **PAC5** probe and these mutant proteins which demonstrated that only mutation of Asp49 led to loss of binding which belonged to only one of these pockets. However, the mutation of other residues critical for the interaction did not affect binding. The molecule was found to increase the translocation of hnRNP A2/B1 from the cytoplasm to the nucleus, as well as modulate kinase signaling involving TBK1 and IRF3. However, how this involves the RNA‐binding capacity of hnRNP A2/B1 is unclear. Since these pathways are important in viral infection the compound was tested in a SARS‐CoV‐2 mouse model where it provided some protection, albeit at a relatively high dose of 100 mg/kg.

The next inhibitor that was described to be able to inhibit hnRNP A2/B1 was **XI‐011** (Figure 5D) (L. Hu et al. 2023). It was previously found in a cellular high‐throughput screen to identify compounds that increase levels of the tumor suppressor p53 (Berkson et al. 2005). The compound was converted into a set of pull‐down probes used to identify its targets using proteomics, although a non‐binding probe was not included making it difficult to exclude nonspecific effects. hnRNP A2/B1 was enriched for both probes and further investigated as a target of **XI‐011**. Indeed, knockdown of hnRNP A2/B1 led to downregulation of MDMX which is one of the main regulators of p53 degradation. Biolayer interferometry was used to study the affinity of the compound for various truncated protein variants and an affinity of 4.6 μM was measured for RRM1. However, affinities of 15.4 and 12.6 μM were measured for RRM2 and the prion‐like domain, respectively. Especially binding to the latter domain is surprising as it is disordered and therefore structurally unrelated to the RRMs, making it unclear whether real binding is observed in this experiment.

Although both **PAC5** and **XI‐011** were predicted to bind to the RNA‐binding interface of hnRNP A2/B1 RRM1, no experimental evidence was provided that either could inhibit RNA‐binding.

### 1.7 hnRNP A18

The RBP hnRNP A18, also known as cold‐inducible RBP, has been reported to be overexpressed in various cancers. It binds mRNA sequences involved in cancer progression that contain its 51‐nucleotide recognition sequence in their 3′UTR (E. T. Chang et al. 2016; R. Yang et al. 2010). By doing so, it increases their translation through recruitment of the translation initiation factor eIF4G under cellular stress. It contains a single RRM domain followed by an unstructured RGG domain (Figure 5B).

An inhibitor for hnRNP A18 was developed by using the computational Site Identification and Ligand Competitive Saturation technique (Solano‐Gonzalez et al. 2021). The crystal structure of the single RRM domain of hnRNP A18 was used for this method that simulates the protein in a box filled with a mixture of various solvent like molecules. The spatial orientation of the functional groups from these molecules binding to the target protein are then converted into a FragMap that describes a pharmacophore, in this case targeted to the RNA‐binding site. Comparison of the FragMap with 780,000 commercially available molecules led to the identification of 40 compounds that matched the spatial calculated pharmacophore and had desirable properties for bioavailability. NMR analyses of these substances indicated that 27 of them induced chemical shift changes in residues involved in RNA‐binding. Four structurally similar compounds were found to have IC_50_ values in an RNA‐competitive FP assay ranging from 2.9 to 15 μM, while none were able to inhibit hnRNP A1 or F. Further selectivity analysis was performed by evaluating the inhibition of the growth of cancer cell lines in comparison to the same cell lines that had hnRNP A18 knocked down. For all compounds, an inhibitory effect was only observed when hnRNP A18 was present. Three of the compounds were evaluated for intracellular target engagement using a CETSA protocol, and although there is indication that they stabilize the target protein, the determined curves are incomplete and therefore difficult to interpret. RIP was used to evaluate the inhibition between hnRNP A18 and one of its RNA targets (CTLA‐4). All compounds showed significant competition, but none completely abolished binding even at high concentrations (200 μM). When tested on three cancer cell lines the most potent compound **508,411** (Figure 5E), as determined in the competition FP assay, had nearly no effect. The less potent **7,646,184** (Figure 5E) caused a strong reduction in viability, while a normal epithelial cell line was unaffected.

### 1.8 La

Several families of La‐related proteins (LARPs) exist, and each has specific functions. Their structure is characterized by the La‐module, which consists of the La motif domain followed by an RRM domain and for several LARPs this is followed by another RRM (Maraia et al. 2017). The defining member La (also known as SS‐B or LARP3) has such a dual RRM structure (Figure 5C) and has been connected to cancer as well as HBV infection (Ehlers et al. 2004; Stavraka and Blagden 2015). La can upregulate known oncogenes such as MDM2 and Cyclin D1, but these effects seems to cancer type specific (Sommer, Dittmann, et al. 2011; Sommer, Rossa, et al. 2011).

To identify inhibitors of La, Sommer et al. performed an FP‐based high‐throughput screen using the full length protein and a fluorescently labeled RNA (Sommer et al. 2017). From 11,520 tested compounds they identified two molecules that had IC_50_ values below 10 μM. Although **C6** (Figure 5F) was able to inhibit the protein‐RNA interaction in an EMSA assay with a similar IC_50_, **C2** required much higher concentrations in the millimolar range. When using only RRM1 and 2 in the EMSA assay, the **C6** compound was still able to inhibit RNA‐binding with similar potency as compared to the full‐length protein. Analogues of **C6** with altered ester substituents (Figure 5F) were found to be approximately fourfold more potent in this EMSA assay. Nonetheless, when tested for cellular activity it was found that **C6** was toxic to both cancerous and healthy cells and therefore the **C2** analogue **C2.01** (Figure 5F) was further studied as it did not have toxicity issues. However, its cellular activity in the mid micromolar range was far higher than the millimolar activity observed in the EMSA assay suggesting that this activity might not be La related.

### 1.9 Tar DNA‐Binding Protein 43

**FIGURE 6:**
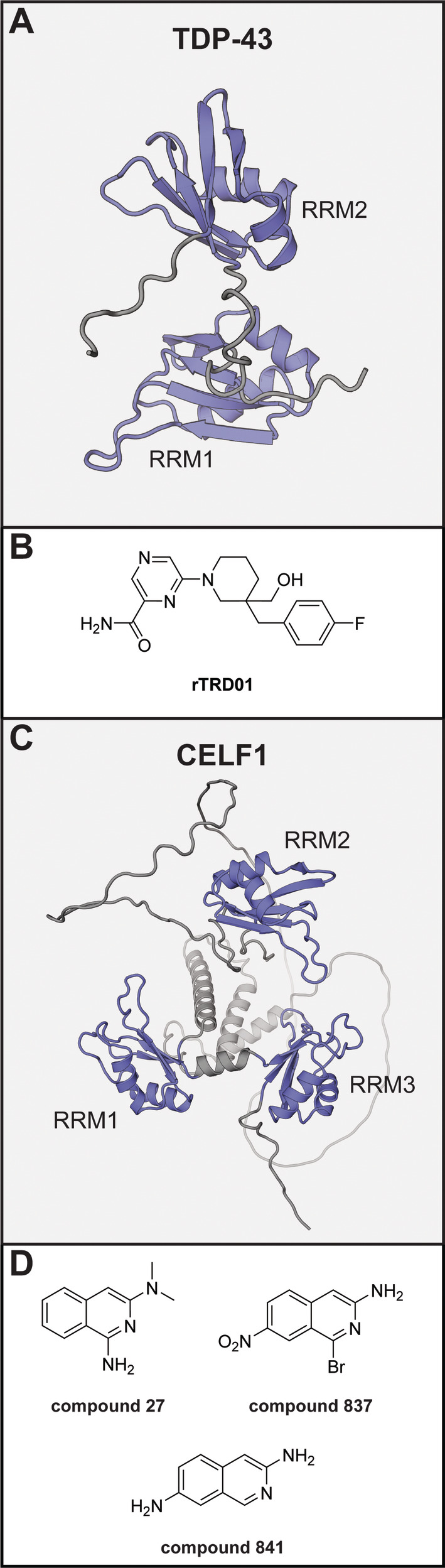
(A) AlphaFold model of TDP‐43 (residues 83–295). (B) Chemical structure of TDP‐43 inhibitor. (C) AlphaFold model of full length CELF1. (D) Chemical structures of CELF1 inhibitors. For (A) and (B), the RRM domains are shown in blue, unstructured domains in gray.

TDP‐43 contains two RRM domains and regulates various aspects of mRNA processing (Figure 6A) (Jo et al. 2020). It is highly conserved and plays a major role in the development of various neurodegenerative diseases. The pathology is caused by accumulation of TDP‐43 aggregates and up to 97% of all ALS patients show such inclusions (de Boer et al. 2021). Similar observations were made in approximately 45% of patients with frontotemporal lobar degeneration, making TDP‐43 an attractive target for therapies to treat these diseases.

Although a few molecules were reported to reduce TDP‐43 aggregation, it was not reported whether they target any of the RRMs (Prasad et al. 2016). An exception to this is the small molecule **rTRD01** (Figure 6B) that was designed to do so and identified via a *in silico* docking screen against RRM1 (François‐Moutal et al. 2019). STD‐NMR validated the binding which was further confirmed by MST measurements using both RRM domains which indicated an affinity of 89.4 μM. An AlphaScreen assay was then used to measure the inhibition between the TDP‐43 RRM domains and two different RNA sequences. Although the compound did show some activity, very high concentrations (1 mM) only led to approximately 50% inhibition. **rTRD01** was further evaluated in a *Drosophila melanogaster* ALS model and found to be able to revert the phenotype in a TDP‐43^G298S^ mutant back to the level of the wild‐type protein.

### 1.10 CELF1

CELF1 is an RBP that was found to play a role in a variety of cardiovascular diseases including myotonic dystrophy and dilated cardiomyopathy, but also in liver fibrosis (K.‐T. Chang et al. 2017; Kuyumcu‐Martinez, Wang, and Cooper 2007; X. Wu et al. 2016). It has three RRM domains (Figure 6C) and generally binds GU‐rich RNA sequences and by doing so regulates splicing, translation and decay of its target mRNAs (Tsuda et al. 2009). One mode of action by which CELF1 contributes to heart disease is by binding and degrading the connexin 43 mRNA which is required for proper contractility (K.‐T. Chang et al. 2017). Therefore, inhibitors could block its aberrant mRNA regulation and provide a therapeutic strategy in the above named diseases.

An inhibitor (**compound 27**, Figure 6D) of CELF1 was found via a virtual screening approach where 100,000 compounds were docked into the RNA‐binding site of RRM2 (Tan et al. 2022). A competitive FP assay, using a truncated protein covering RRM1 and 2 and a labeled GU‐rich RNA, was used to determine an IC_50_ of 22.99 μM which was further confirmed using an EMSA assay. The binding site of **compound 27** was identified through molecular docking and several mutations in this site were made for verification. The affinity for these mutants was measured by isothermal titration calorimetry and a loss of affinity was observed when the critical lysine 117 was mutated to alanine, although mutation of two other residues that were predicted to be essential for binding did not affect the interaction. Selectivity was evaluated against a single protein, in this case HuR, for which the compound did not show any inhibition. Hepatic stellate cell activation is a key step liver fibrosis and was modulated by **compound 27** by stabilizing *IFN‐γ* RNA which is normally degraded by CELF1 as well as downregulating *ACTA2* and *COL1A1* mRNA. Use of the compound in a mouse model of liver fibrosis reduced the fibrotic surface and improved levels of biomarkers at a 3 mg/kg maximum dose. However, the pharmacokinetic/pharmacodynamic analysis demonstrated that the blood concentration rapidly drops below 1 μM, which is well below the *K*_i_ values for RNA inhibition, making it unclear whether these effects are related to CELF1 inhibition. Structural optimization led to **compounds 838** and **841** with *K*_i_ values of 12.28 and 1.52 μM, respectively, in the competitive FP assay. A pull‐down assay using a biotinylated GU‐RNA (recognized by CELF type proteins) was used to evaluate selectivity within the family. Although **compound 27** was not selective, **compound 841** was more selective for CELF1 only inhibiting CELF2 moderately, warranting further evaluation of this compound.

### 1.11 Alternative Strategies to Target RRM Domains

Various alternative strategies to target RRM domains have been explored including small molecules that do not directly target the RNA‐binding site of the domain, covalent inhibitors, and inhibitors of other modalities such as oligonucleotides and peptides. Each strategy has its own advantages and disadvantages, but since they were used to target other proteins than the ones described above it is not possible to make a direct comparison. Known examples of such alternative RRM targeting strategies will be described in this section.

### 1.12 U2AF

**FIGURE 7:**
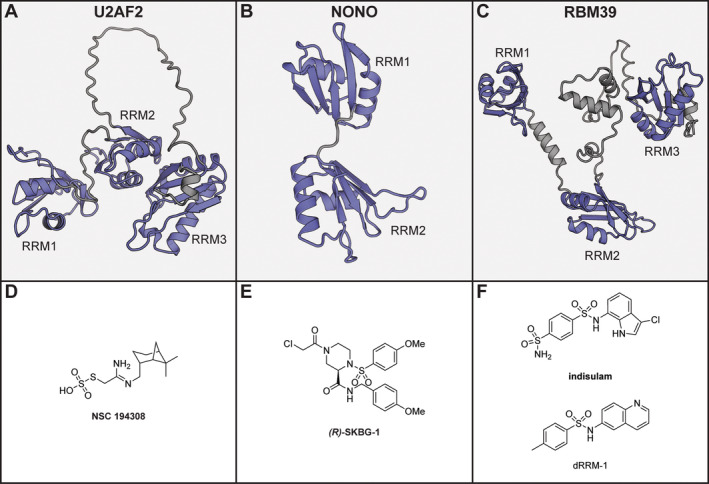
(A) AlphaFold model of U2AF2 (residues 149–475). (B) AlphaFold model of NONO (residues 74–227). (C) AlphaFold model of RBM39 (residues 153–530). (D) Chemical structure of U2AF2 inhibitor. (E) Chemical structure of NONO inhibitor. (F) Chemical structures of RBM39 inhibitors. For (A)–(C), the RRM domains are shown in blue, unstructured domains in gray.

The U2AF complex is responsible for nucleation of spliceosome assembly and consists of a heterodimer of U2AF1 and U2AF2 which recognizes polypyrimidine tracts and AG‐dinucleotides (Guth et al. 2001; Singh, Valcárcel, and Green 1995; S. Wu et al. 1999). Mutations in U2AF1 as well as in various other splicing factors (SF3B1, SRSF2, and ZRSR2) are observed in hematological cancers and have generated interested in inhibitors that correct the splicing errors they cause (Patnaik et al. 2013). During the initial binding events for spliceosome assembly a ternary complex is formed of U2AF1, U2AF2, and SF1 which is then replaced by SF3B1. U2AF2 is a three RRM domain protein (Figure 7A) and has been explored as a therapeutic target based on the success of small molecule SF3B1 inhibitors in the treatment of leukemia.

Using an FP HTS assay, Chatrikhi et al. identified the small molecule **NSC 194308** (Figure 7D) (Chatrikhi et al. 2021). The molecule stabilized the interaction between the U2AF1‐U2AF2‐SF1 complex and an RNA covering a splice site of the *DEK* oncogene with an EC_50_ of 100 μM. **NSC 194308** could inhibit spicing in vitro, while docking and biophysical analysis, with a construct containing the U2AF2 RRM1 and 2, suggested that the small molecule binds in between the two RRMs. This was further confirmed using the individual domains or point mutations in FP assays as well as by docking studies by another group (Rozza, Janoš, and Magistrato 2023). Cellular splicing assays were then used to show that U2AF2‐sensitive transcripts were alternatively spliced upon **NSC 194308** treatment. Interestingly, HEK239T cells and leukemia cells with an U2AF1 mutant phenotype were more sensitive to **NSC 194308** toxicity than WT cells (Chatrikhi et al. 2021). Although binding to other RRM‐containing proteins was not investigated, the allosteric binding site is less conserved and might therefore lead to higher selectivity.

### 1.13 NONO

Another example of a compound binding in between two RRM domains was found by Kathman et al., who identified the compound **(*R*)‐SKBG‐1** as an inhibitor of the RBP NONO (Kathman et al. 2023). NONO is part of the Drosophila behavior/human splicing family of proteins which can bind both RNA and DNA via conserved dual RRM domains (Figure 7B) (Knott, Bond, and Fox 2016). They have a variety of functions including the regulation of transcription, as well as post‐transcriptional RNA processing steps such as splicing, polyadenylation and stabilization. To regulate splicing, NONO is known to interact with the spliceosome, but can also bind the 5′ splice site directly (Kameoka, Duque, and Konarska 2004; Peng et al. 2002). NONO plays a role in various cancers either as an oncogene on its own or as a fusion to various other proteins (Feng et al. 2020). The compound **(*R*)‐SKBG‐1** was not identified by directly targeting NONO itself, but via two phenotypic assays evaluating androgen receptor (AR) expression. The first assay monitored the mRNA levels of full‐length AR as well as the isoform V7, and the second assay used high‐content imaging to determine the protein levels of AR and the cytotoxicity of the tested compounds. From both screening assays, a single hit was obtained from a library of 500 in‐house developed electrophilic compounds with cysteine reactive warheads for covalent binding to the targets. Structural optimization, by substitution of the two benzyl groups, led to the more potent **(*R*)‐SKBG‐1** (Figure 7E) and the enantiomeric **(*S*)‐SKBG‐1** was found to be completely inactive. Using both compounds in a cysteine‐directed activity‐based proteome profiling experiment, the target NONO was identified. The cysteine residue 145 was found to be engaged by the active compound, but not the inactive enantiomer. Cysteine 145 is found in between the two RRM domains of NONO and very close to residues expected to be involved in RNA‐binding. In NONO knockout cells, the compound had no effect on AR expression, but when NONO was reintroduced the activity of the compounds was restored. This was not the case in cells expressing a mutant without the cysteine (C145S), indicating that the covalent interaction with NONO was a requirement for activity. To investigate RNA binding, the authors made use of enhanced UV cross‐linking and immunoprecipitation experiments, which demonstrated that **(*R*)‐SKBG‐1** was able to stabilize the interaction between NONO and its target mRNAs. Splicing was also affected by the compound, although in‐depth analysis of the RNA‐seq data indicated that this was not caused by NONO directly, but rather by affecting the expression of other splicing factors.

### 1.14 RBM39

RBM39 is a serine/arginine rich splicing factor that is found as part of early spliceosome complexes and has three RRM domains (Figure 7C) (Campagne et al. 2023). The first two regulate RNA‐binding while the third has evolved to mediate only PPIs, and is therefore often referred to as a U2AF2‐homology motif (UHM) (Campagne et al. 2023; Stepanyuk et al. 2016). It has received significant attention as a key regulator of splicing events and is upregulated in various cancers (R. Zhang, Wang, et al. 2022). The attention for this target increased further after the discovery of the mode of action of several sulfonamide‐based molecules, which act as molecular glues between RBM39 and the ubiquitin ligase DCAF15 (Bussiere et al. 2020; Faust et al. 2020; Han et al. 2017). The sulfonamides stabilize the interaction between these two proteins, which leads to ubiquitination of RBM39 and subsequent degradation by the proteasome (Han et al. 2017). Several RBM39 mutations (Met265, Gly268, Glu271, and Pro272) were found to render cells resistant to the sulfonamide **indisulam** (Figure 7F), which all mapped to the first helix of RRM2, leading to the hypothesis that this could be the binding site. Structural investigation of **indisulam** in complex with DCAF15‐DDB1‐DDA1 and RBM39, using both x‐ray crystallography and cryogenic electron microscopy (cryo‐EM), confirmed this hypothesis (Bussiere et al. 2020). Identical binding modes were observed for two analogues of **indisulam** that have a similar mode of action (Faust et al. 2020). Based on the structure as well as the resistance mutations, Bussiere et al. performed bioinformatic analysis to identify other proteins that had similar helices and could potentially be degraded by **indisulam** (Bussiere et al. 2020). Only RBM23 was found as a match, and proteomics analysis of HCT116 cells after **indisulam** treatment, indeed indicated that RBM39 and 23 were the most strongly downregulated. Although clinical trials demonstrated that these compounds are well tolerated, the effects in cancer were minimal and optimization would be necessary (Xu, Nijhuis, and Keun 2022). Most of these trials were run before the mode of action was determined, but now that this information is available optimized analogues can potentially be developed and although this is promising, it is challenging to identify molecular glues in targeted screening assays. Nonetheless, Hanzl et al. recently developed a screening platform that allows the targeted search of molecular glues by inhibiting auto‐degradation of the E3 ligase of interest (Hanzl et al. 2023). The screen identified a novel sulfonamide (**dRRM‐1**) as a molecular glue for the DCAF15‐RBM39 interaction, thereby demonstrating that screening is indeed possible. Although none of these molecules inhibit the interaction RBM39 or 23 with RNA directly, the degradation of these proteins reduces the occupancy on their target RNAs.

### 1.15 PTBP1

**FIGURE 8:**
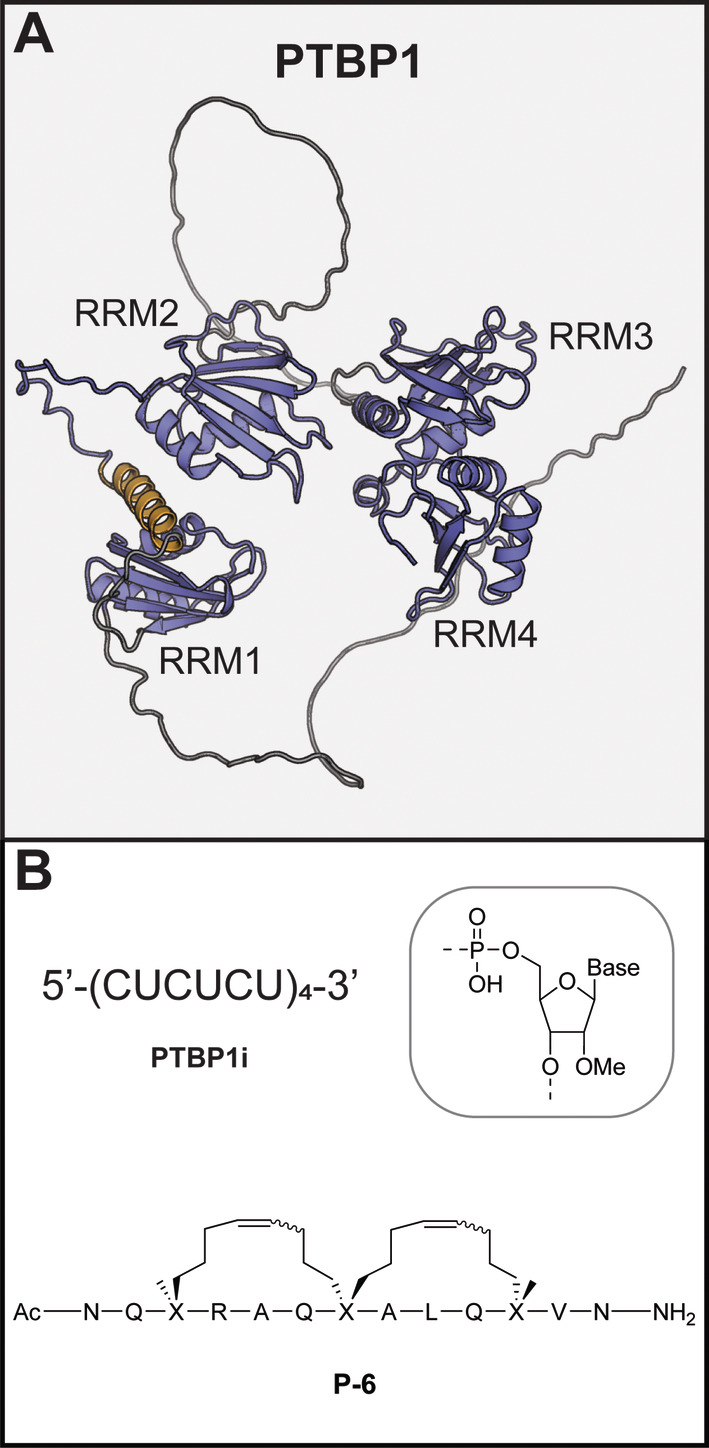
(A) AlphaFold model of full length PTBP1. The RRM domains are shown in blue, unstructured domains in gray, and the extra helix of RRM1 in orange. (B) Chemical structures of PTBP1 inhibitors.

PTBP1 has four RRM domains, and as the name implies binds polypyrimidine rich sequences (Figure 8A) (Dorn et al. 2023). Like hnRNP A2/B1, it is part of the family of hnRNPs (it is also known as hnRNP I) and is well studied for its role as a splicing factor (Geuens, Bouhy, and Timmerman 2016; Llorian et al. 2010). PTBP1 is overexpressed in a wide variety of cancers making it an attractive therapeutic target (Huang et al. 2022; Yu et al. 2023), but also plays a role in cardiovascular and neurodegenerative diseases mainly via regulating splicing (Caruso et al. 2017; T. Kim et al. 2014).

The first PTBP1 inhibitor described (**PTBP1i**, Figure 8B) is an oligonucleotide which is fully methylated on all its 2′ hydroxyl groups to increase its stability. It has four repeats of an RNA sequence that was previously described to be recognized by PTBP1 (CUCUCU) to facility high affinity. The oligonucleotide acts as a decoy by occupying the RNA‐binding sites of the RRMs to compete with endogenous RNAs. Pull‐down experiments using a biotinylated variant of the oligonucleotide and nuclear extracts from HEK293 cells, showed that several other RRM containing proteins were not bound (RBFOX2, SRSF1, SRSF5, and SRSF6). A more exhaustive proteomics analysis of the enriched proteins indicated that a variety of RBPs was bound, including various ribosomal proteins and splicing factors. Nonetheless, **PTBP1i** was able to efficiently modulate splicing events known to be regulated by PTBP1, after transfection into MDA‐MB‐231 breast cancer cells, and inhibit proliferation and colony formation.

Our group developed an alternative approach to inhibiting PTBP1, by exploiting the extra helix of RRM1 as a unique structural feature (Figure 8A) (Schmeing et al. 2023). The helix was previously found to be dynamic in nature and NMR studies showed it to be largely unfolded in the absence of RNA (Maris et al. 2020). When PTBP1 binds RNA, the helix folds and packs to the side of the beta sheet and the first RRM helix opposite to the RNA‐binding site. Although the exact role of the helix is still under investigation it was shown to be important for RNA binding and to be involved in sensing of the secondary structure of the bound RNA as well as positioning of RRM2 (Damberger et al. 2024; Dorn et al. 2023; Maris et al. 2020). We hypothesized that occupying its binding site on RRM1 with a synthetic peptide could lead to allosteric inhibition of RNA binding without directly targeting the RNA binding interface. To mimic the helix, a peptide was prepared using a variation of the hydrocarbon stapling strategy, known as peptide stitching, to produce the inhibitor **P‐6** (Figure 8B) (Hilinski et al. 2014). Here, three amino acids that are not involved in binding to the target are replaced with amino acids with terminal alkenes which are connected together using a dual ring‐closing metathesis reaction forming a “stitch” that locks the peptide in a helical conformation. **P‐6** was able to inhibit RNA binding of a PTBP1 variant spanning RRM1 and 2 and crystallography analysis confirmed it occupied the binding site of the helix it was designed from. The stitching also provided the peptide with cell‐permeability and when used in HEK293T cells, the compound was able to modulate the splicing of exon 10 of PTBP2, which is regulated by PTBP1. Selectivity was evaluated against two other proteins that have dual RRMs (SRSF1 and hnRNP A2/B1) and neither was inhibited by **P‐6**. Although the selectivity study is limited, the binding site targeted by the peptide is less conserved than the RNA‐binding site which could lead to higher selectivity.

## 2 Conclusion

Across all the RRM targeting ligands discussed here, it is apparent that most tend to have affinity for the RRM or inhibitory potency toward RRM–RNA interaction in the micromolar range. The general mediocre potency likely reflects the lack of well‐defined pockets on the RNA‐binding surface of RRMs for effective binding. More successful approaches include the NONO inhibiting **(*R*)‐SKBG‐1** and the RBM39 targeting sulfonamides. Both have a novel mode of action that highlights the concept of addressing the RRM domain in other ways than directly targeting the RNA‐binding surface. The currently described small molecules that do target the RNA‐binding site have a high structural variation, making it hard to judge what chemical properties are required for an inhibitor to target this challenging domain effectively. Many of the small molecules tend to be very flat and have structural features that overlap with those found in pan assay interference compounds (PAINS), which are known to falsify assay read‐outs (Baell 2016). Furthermore, several of the ligands have known activities against other targets which can confound cellular studies. Such compounds should be avoided when designing screening libraries to prevent false positive hits.

Besides potency, selectivity is another major challenge in targeting RBPs via their RRM domain. This is due to the RNA‐binding residues of the RRM domain being highly conserved and the RRM being one of the most abundant RNA‐binding domains of the human proteome. The thermal proteome profiling studies on **Ro‐08‐2750** have highlighted that targeting the RNA‐binding interface has limited success in generating selective molecules (Walters et al. 2023). Applying such label‐free proteomics studies to other RRM targeting ligands could be highly informative to see how and if selectivity can be achieved. It is highly recommendable to perform such studies for new molecules, so that induced phenotypic changes can be correlated with their biochemical activity. The necessity for studying this aspect is highlighted by the fact that many of the described inhibitors require lower concentrations to achieve a read‐out in cellular assays than their biochemically measured affinities. These results suggest that those cellular effects are caused by the interaction of the molecules with other targets, or even more nonspecific events such as DNA intercalation or membrane perturbation. To avoid the identification of such molecules in the first place, it would be useful to screen libraries of sufficient diversity and avoid those with known activities. Furthermore, avoiding the RNA‐binding site of an RRM domain provides the opportunity to find more selective molecules. Specifically, composite pockets between RRMs and other proteins as well as PPI sites on RRM domains are likely to be much less conserved and can provide binding sites for selective inhibition. However, composite pockets are hard to identify and are often found serendipitously, making the development of a targeted screening assay challenging. PPI sites on the other hand would be more amenable for targeted screening (Egbert et al. 2019). The inhibitors of UHM‐domains which are similar to RRMs, but only bind protein, highlight this possibility (Jagtap et al. 2016, 2020). However, a comparative study on the selectivity of such inhibitors also indicated that this is challenging to achieve for this domain, highlighting the need for the identification of further sites (Yuan et al. 2023).

Finally, another challenge in targeting RBPs that contain RRM domains is that they often contain more than one RRM or additional RNA‐binding domains of another type. The question arises whether the inhibition of a single domain is sufficient for the desired effect.

In conclusion, to effectively inhibit RBPs via their RRM domains, creative approaches are required, as well as a thorough investigation of the mode of action and selectivity of a hit molecule. Sites, other than the RNA‐binding interface of the RRMs, should be explored to overcome the above‐mentioned challenges and to do so we believe it is necessary to look beyond classical rule‐of‐five molecules. Modern selection methods could allow the generation of selective molecules from a variety of classes such as aptamers, cyclic peptides, or small proteins. The larger size of these molecules creates more interaction possibilities, not only improving affinity, but also selectivity. To overcome some of the challenges described above, the use of composite pockets is a solution which is exemplified by the RBM39 targeting sulfonamides. Degradation of the target protein, as these sulfonamides induce, is especially attractive since the occurrence of other RNA‐binding domains then becomes irrelevant. Indeed, RBM39 has three RRM domains but can still be inhibited through this mechanism by binding to only one. The development of advanced screening methods for the detection of targeted protein degraders for RRM containing proteins is therefore necessary. By using these novel, and more advanced, approaches we believe that these challenging targets can be addressed with success.

## Author Contributions

**Stefan Schmeing:** conceptualization (equal), writing – original draft (lead), writing – review and editing (supporting). **Peter 't Hart:** conceptualization (equal), supervision (lead), writing – original draft (supporting), writing – review and editing (lead).

## Conflicts of Interest

The authors declare no conflicts of interest.

## Related Wires Articles

The La and related RNA‐binding proteins (LARPs): Structures, functions, and evolving perspectives.

Classification and function of RNA‐protein interactions.

The roles of hnRNPA2/B1 in RNA biology and disease.

## Data Availability

The authors have nothing to report.
